# Essential oils: a systematic review on revolutionizing health, nutrition, and omics for optimal well-being

**DOI:** 10.3389/fmed.2024.1337785

**Published:** 2024-02-16

**Authors:** Camila Pezantes-Orellana, Fátima German Bermúdez, Carmen Matías De la Cruz, José Luis Montalvo, Andrea Orellana-Manzano

**Affiliations:** ^1^NovaVita, Guayaquil, Ecuador; ^2^Laboratorio para Investigaciones Biomédicas, Facultad de Ciencias de la Vida, Escuela Superior Politécnica del Litoral (ESPOL), Guayaquil, Ecuador

**Keywords:** essential oils, nutrition, omics, bioactive compounds, well-being

## Abstract

**Purpose:**

Essential oils from various plants have diverse therapeutic properties and are researched extensively. They have applications in medicine, aromatherapy, microbiology, agriculture, livestock, and the food industry, benefiting the population.

**Methods:**

This systematic review followed the PRISMA verification protocol. The study focused on the anti-inflammatory effects, nutraceutical properties, antioxidant and antibacterial activity of essential oils in lemon, orange, cumin, cinnamon, coriander, rosemary, thyme, and parsley. We also looked at their presence in the diet, their effect, their mechanism of action on health, and the most important active compounds. The search was conducted in the PubMed database for the last 12 years of publications, including *in vitro*, *in vivo*, and online cell model tests.

**Results:**

Essential oils have been shown to have multiple health benefits, primarily due to their antimicrobial and anti-inflammatory effects. The mechanism of action of cinnamon oil alters bacterial membranes, modifies lipid profiles, and inhibits cell division, giving a potential benefit in protection against colitis. On the other hand, a significant improvement was observed in the diastolic pressure of patients with metabolic syndrome when supplementing them with cumin essential oil. The antimicrobial properties of coriander essential oil, especially its application in seafood like tilapia, demonstrate efficacy in improving health and resistance to bacterial infections. Cumin essential oil treats inflammation. Parsley essential oil is an antioxidant. Orange peel oil is antibacterial, antifungal, antiparasitic, and pro-oxidative. Lemon essential oil affects mouse intestinal microbiota. Thyme essential oil protects the colon against damage and DNA methylation. Carnosic acid in rosemary oil can reduce prostate cancer cell viability by modifying the endoplasmic reticulum function.

**Conclusion and discussion:**

Essential oils have many therapeutic and antiparasitic properties. They are beneficial to human health in many ways. However, to understand their potential benefits, more research is needed regarding essential oils such as coriander, parsley, rosemary, cumin, and thyme. These research gaps are relevant since they restrict understanding of the possible benefits of these crucial oils for health-related contexts.

## 1 Introduction

Essential oils (EOs) are fragrant extracts obtained from various plants. Their composition varies depending on the plant species from which they are extracted. It is estimated that more than 200 compounds may be present in these oils. In recent years, essential oils have gained significant popularity in various industries, such as aromatherapy, food flavoring, and natural pharmacological treatments, due to their numerous uses, primary components, and respective properties. Consequently, several applications have been studied, including their antimicrobial, anti-inflammatory, analgesic, and antioxidant properties (1).

The main bioactive compounds of EOs are terpenes and terpenoids, which are responsible for the biological activities mentioned above (2). So, the properties of EOs contribute to the prevention of diseases through different mechanisms of action. *In vitro* studies are generally carried out, and it has been shown that the anti-inflammatory components of EOs inhibit free radicals that can generate mutations alone. In the DNA. Likewise, when there is prolonged oxidative stress, an excessive accumulation of reactive oxygen species (ROS) can trigger chronic disorders such as metabolic syndrome, cardiovascular diseases, diabetes, and even cancer for the same reason that there are mutations in the DNA. EOs rich in polyphenols and their antioxidant properties act as therapeutic agents for these diseases (3).

Essential oils are used in healthcare to treat specific diseases or health conditions. They can be used to alleviate symptoms associated with conditions such as Alzheimer’s, cardiovascular diseases, sleep/stress disorders, and pain during childbirth (4). Research has shown that rosemary essential oil has potential anticholinesterase inhibitory and antioxidant effects that may help protect the brain from chronic anticholinesterase diseases such as Alzheimer’s. However, more studies are needed to determine the adverse effects or benefits. Additionally, aromatherapy has been found to improve cognitive function in patients with such conditions (5).

Antimicrobials are used for various purposes, including medical use as antiviral agents, immunomodulators, and antibiotics. They are also used as food preservatives due to their antimicrobial and antioxidant properties, which help counteract skin infections and, in addition to preventing food spoilage, are used to combat microorganisms that could be transmitted through food (4). According to Swamy et al. (6), lemon has antiviral properties against the influenza virus, while cinnamon is effective against enterobacteria.

Essential oils can affect bacteria differently, depending on their chemical components. Some oils can kill the bacteria (bactericidal action), while others can only slow their growth (bacteriostatic action). Essential oils can also affect cellular processes, such as nutrient processing, molecule synthesis, and regulation of biological processes between cells. Many plants with these characteristics are still being studied, and more findings are expected as they are widely used. These plants include cinnamon, thyme, rosemary, lemon, orange, cumin, and parsley (4). This systematic review identifies literature from the past 10 years to highlight their uses and effects on health, diet, microbiota, and mechanism of action.

## 2 Methods

This systematic review follows the PRISMA (Preferred Reporting Items for Systematic Reviews and Meta-Analysis) verification protocol (7) with keywords in the search strategy as Essential oils, Nutrition, Omics, Bioactive Compounds, Well-being, properties, Application in Foods previously published experimental and/or clinical case trials in mouse models and/or cell lines and studies in human participants were used along with the use of search operators AND, OR. The study focuses on the anti-inflammatory effects, nutraceutical properties, antioxidant, and antibacterial activity, presence in food, effect and mechanism of action on health, and the most important active compounds of essential oils such as lemon, orange, cumin, cinnamon, coriander, rosemary, thyme, and parsley by searching for articles conducted in a cell line (*in vitro*), mouse model, *in vivo* specifically in PubMed database.

The study selection process was conducted in two stages. In the first stage, three authors independently reviewed the titles and abstracts of the studies based on the inclusion and exclusion criteria. They identified the studies that met the inclusion criteria and excluded the ones that did not. In the second stage, two authors thoroughly screened all the full articles and excluded the studies that did not meet the inclusion criteria. We developed custom search strategies for the PubMed bibliographic database. The search was conducted directly and included all articles published within the last 12 years without language restrictions. Additionally, we thoroughly examined the reference lists of the selected articles to identify any relevant research that may have been missed during the electronic database search. Three authors gathered the required data from the reports that had been selected. For all incorporated studies, the following details were recorded: author(s), year of publication, type of essential oil, its impact on health, its effects on health, and its presence in the diet. The authors independently reviewed all full articles.

## 3 Eligibility criteria

### 3.1 Inclusion and exclusion criteria

Studies were considered eligible if they met the following criteria: (1) retrospective experimental and clinical studies in human subjects and mouse models and/or cell lines, (2) studies investigating the health benefits of the essential oils described above, (3) studies with quantitative and qualitative data, (4) studies without any language restrictions, (5) systematic reviews, and (6) manuscripts in journals with an impact index.

The following studies were excluded: (1) not meeting the objectives of the article, (2) university theses, (3) not meeting the search criteria, (4) book chapters or books, (5) review articles published more than 12 years ago, and (6) studies not found in the database described above.

### 3.2 Search strategies

To refine the search, specific keywords such as “Essential oils” “Nutrition” “Omics” “Bioactive compounds” “Food application “Wellness” “properties” “mouse models ““experimental” “clinical” “cell lines” were used together with the use of search operators (Nutrition* OR “dietary” OR “nutritional* content” OR “macronutrients” OR “macronutrients” OR “diet”) AND (“Essential oils*” OR “volatile oils* “OR “Extracted oils* OR “Aromatherapy oils*” OR “Plant oils* “) AND (“Essential oils*” OR “Plant oils*” OR “macronutrients” OR “diet”) AND (“Essential oils*” OR “volatile oils* “OR “Extracted oils* Aromatherapy oils*” OR “Plant oils*) AND (Omics* OR “Microbiota*” OR “Genomics*” OR” Metabolomics*”) AND (Bioactive compounds* OR “Functional ingredient” OR “Major active compound *” OR “Active substance” OR “Health-promoting compound*”) AND (Food application * OR “Application in Food Safety “OR “Application in Food Industry”) AND (Wellness* OR “health “) AND (properties* OR “Aspects “OR “Traits”) performed in electronic databases such as Pubmed, ScienceDirect and Scopus.

## 4 Results

### 4.1 Study selection

**Figure 1:**
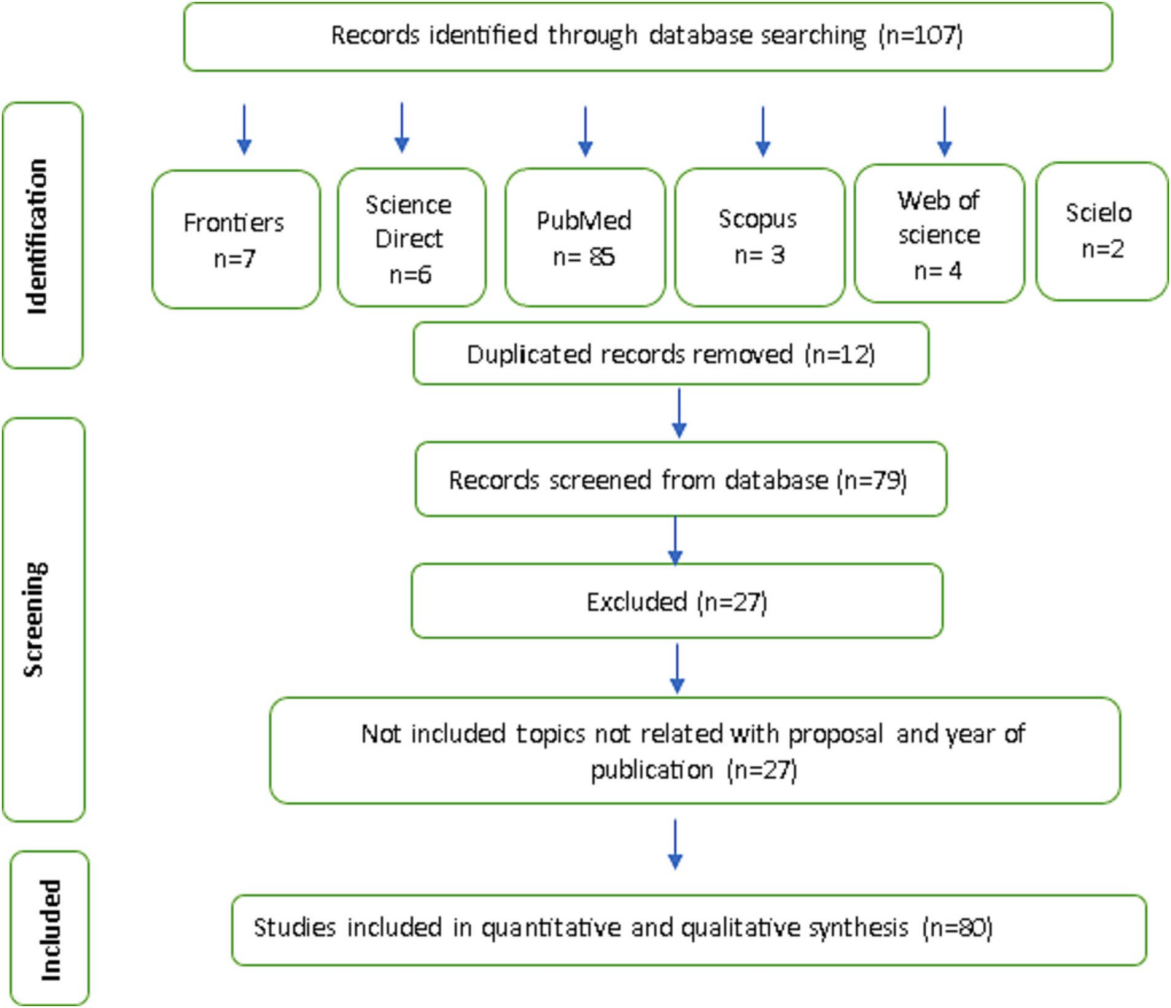
Flow diagram of literature search and selection criteria for essentials oils from PRISMA.

In the first phase of study selection, 107 citations were found in the PubMed electronic database. After thoroughly reviewing the abstracts, we excluded 27 articles with 12 duplicates. We then identified 85 additional articles through PubMed and reviewed their full text. Thus, this study included 80 references (Figure 1).

### 4.2 Study characteristics

The table below summarizes the key features of the studies analyzed in this research. These studies were conducted in different countries and published between 2011 and 2023 in both English and Spanish. It was observed that the number of publications increased significantly from 2019. All the selected studies were focused on essential oils, with most of them investigating the properties of these oils, such as their antimicrobial and anti-inflammatory effects. The studies have produced significant findings on the benefits of using essential oils, including inhibiting pathogenic microorganisms, preventing radical formation, acting as natural antioxidants and antimicrobial agents, exhibiting anticancer effects in the intestinal microbiota, and preserving food naturally.

Table 1 shows the type of food used, the analysis of the major active constituent identified, and relevant results in the essential oils performed *in vivo* or *in vitro*. The main groups of constituents were in cinnamon essential oil cinnamaldehyde, coriander essential oil β-linalool, camphor, geranyl acetate, and cymene; in cumin essential oil cumin aldehyde, and parsley essential oil myristicin, apiole, α-pinene, and β-pinene. Table 2 shows the most predominant bioactive compounds, their health effects, and tentative mechanisms. The main compounds in lemon and orange essential oils are limonene. In cinnamon essential oil, cinnamaldehyde; in Cilantro essential oil, linalool; in Parsley essential oil, myristicin; in Rosemary essential oil monoterpenes; in Thyme essential oil, thymol with antioxidant activity, antimicrobial, inflammatory activity, and innate immune responses.

**Table 1:** Essential oils and their used as food.

	Essential oil	Food use type	Type of study / cell line model (*in vitro*), mouse model, *in vivo*	Study name	Relevant results	Major active compound	Reference
1	Cinnamon	This study was carried out to evaluate the effects of cinnamon (CO), rosemary (RO) and a mixture (MO) of these plants oil on egg production, feed intake, feed conversion rate, external and internal egg quality, blood serum mineral contents and hatchability traits in different sex.	*in vivo*	Effects of cinnamon and rosemary oils on egg production, egg quality, hatchability traits and blood serum mineral contents in laying quails (*Coturnix coturnix Japonica*).	The addition of cinnamon oil to the diets significantly reduced Feed conversion ratio (FCR).	Cinnamaldehyde (88.2%)	(8)
2	Coriander	Essential Oil for Its Application in Foods	*in vitro*	Antioxidant, antimicrobial and antibiofilm activity of coriander (*Coriandrum sativum L.*) essential oil for its application in foods	The major volatile compounds of the coriander essential oil in the present study were β-linalool 66.07%. Coriander essential oil radical scavenging activity was 51.05% inhibited. Coriander essential oil expressed the strongest antibacterial activity against *B. subtilis* followed by *S. maltophilia* and *Penicillium expansum.* The strongest antibiofilm activity of the coriander essential oil was found against *S. maltophilia*.	β-linalool 66,07%, alcanfor 8,34%, acetato de geranilo 6,91% y cimeno 6,35%	(9)
3	Coriander	Inclusion of coriander oil in tilapia feed to improve the health of tilapia and its resistance to bacterial infections	*in vivo*	Dietary coriander (*Coriandrum sativum L*) oil improves antioxidant and anti-inflammatory activity, innate immune responses, and resistance to *Aeromonas hydrophila* in Nile tilapia (*Oreochromis niloticus*)	Coriander oil with 1% incorporation in feed improves tilapia health and resistance to bacterial infection.	Inalool and geranyl acetate	(10)
4	Cumin	Cumin volatile oils suppressed the activation of neutrophils and might show therapeutic potential for the treatment of neutrophilic inflammatory diseases.	*In vitro*	Anti-Inflammatory and Antimicrobial Volatile Oils: Fennel and Cumin Inhibit Neutrophilic Inflammation via Regulating Calcium and MAPKs	Suppressed the activation of human neutrophils, including respiratory burst and the degranulation induced by formyl peptide receptor agonists fMLF/CB and MMK1 in the human neutrophils (IC50, 3.8–17.2 μg/mL).	Cumin volatile oil (CUMIN) revealed high content of cuminaldehyde (49.9%)	(11)
5	Cumin	The supplementation with cuminum essential oils decreased diastolic blood pressure (BDP) in patients with metabolic syndrome	Randomized and controlled clinical trial	Effects of cumin (*Cuminum cyminum* L.) essential oil supplementation on metabolic syndrome components: A randomized, triple-blind, placebo-controlled clinical trial	56 patients with MetS aged 18–60 years received either 75 mg CuEO or placebo soft gel thrice daily for 8 weeks. DBP was significantly lower in CuEO compared with the placebo group at the end of study (81.41 ± 5.88 vs. 84.09 ± 5.54 mmHg, MD with 95% CI: −3.98 [−7.60, −0.35] mmHg, *p* < 0.05).	Not mentioned	(12)
6	Parsley	Parsley presented the best antioxidant profile	*In vitro*	Chemical Composition, Antioxidant and Antimicrobial Activity of Essential Oils from Organic Fennel, Parsley, and Lavender from Spain	Parsley had the highest phenolic content. Overall, parsley presented the best antioxidant profile, given its highest % of inhibition of DPPH radical (64.28%) and FRAP (0.93 mmol/L Trolox), but had a pro-oxidative behavior by TBARS.	Myristicin (36.15%), apiole (20.97%), α-pinene (15.47%), and β-pinene (10.43%).	(13)

**Table 2:** Actions in the health of the essential oils.

	Essential oil	Health action	Type of study	Study name	Relevant results	Major active compound	Tentative mechanism (how this essential oil has the effect on health)	Reference
1	Lemon	Showed DPPH radical scavenging activity Demonstrated a good inhibitory activity against the pathogenic microorganisms *C. albicans, L. monocytogenes* and *S. aureus.*	Cell line model	Waste *Citrus limon* Leaves as Source of Essential Oil Rich in Limonene and Citral: Chemical Characterization, Antimicrobial and Antioxidant Properties, and Effects on Cancer Cell Viability	It showed DPPH radical scavenging activity (IC50 value = 10.24 mg/mL) and demonstrated a good inhibitory activity against the pathogenic microorganisms *C. albicans, L. monocytogenes* and *S. aureus.* Moreover, the treatment with LLEO significantly affected cell viability and morphology from 25 μM in cancer HeLa and A375 cells, with lower effects on normal fibroblasts and keratinocytes.	Limonene (256.7 ± 2.4 mg/mL), Citral (194.5 mg/mL)	The antimicrobial activity against bacterial species tested were strain dependent. The antioxidant activity is given as IC50 value, which indicates the LLEO concentration required to give a 50% inhibition of the DPPH• radical formation LLEO exhibited good antioxidant activity, with an IC50 value of 10.24 ± 2.8 mg/mL.	(14)
2	Lemon	Antioxidant and antimicrobial activities with its preservative effect against *Listeria monocytogenes*	Inoculated in minced beef meat	*Citrus* lemon essential oil: chemical composition, antioxidant, and antimicrobial activities with its preservative effect against *Listeria monocytogenes* inoculated in minced beef meat	The application of *Cl*EO at a 0.06 and 0.312 mg/g, may open new promising opportunities for the prevention of contamination from and growth of pathogenic bacteria, particularly *L. monocytogenes*, during minced beef meat storage at 4°C. Additionally, during storage period, physicochemical values were higher in control meat than treated meat with *Cl*EO suggesting an efficient antioxidant activity of *Cl*EO.	Limonene (39.74%) and β-Pinene (25.44%).	It is demonstrated, *in vitro* and *in situ*, the efficiency of ClEO as a natural antioxidant and antimicrobial agent.	(15)
3	Lemon/ Grapefruit	Antibacterial activity and antioxidant activity	*In vitro*	The Chemical Composition and Antibacterial and Antioxidant Activities of Five *Citrus* Essential Oils	The LEO and DLEO demonstrated greater antioxidant activity, measured as DPPH and ABTS free radicals compared with other EOs. The high concentration of d-limonene appeared not to be necessarily associated with high antibacterial and antioxidant activity, suggesting that the antibacterial and antioxidant activities might be related to the active component profiles and possibly their synergism effects.	Monoterpenes (d-limonene and less so sesquiterpenes)	The antibacterial activities of the EOs on three bacteria (*Escherichia coli*, *Salmonella* and *Lactobacillus acidophilus*) were tested by measuring the minimum inhibitory concentration (MIC), minimal bactericidal concentration (MBC) and inhibition zone diameter (IZD).	(16)
4	Orange	The EOP and EOPD showed antioxidant activity by reducing metals, and particularly the EOP by also neutralizing free radicals. They partially affected the bacterial growth while strongly inhibiting the biofilm formation and viability of sessile bacteria living in a pre-existent biofilm (Gram-negative and Gram-positive)	*In vitro*	*Citrus sinensis* Essential Oils an Innovative Antioxidant and Antipathogenic Dual Strategy in Food Preservation against *Spoliage Bacteria*	They could represent natural and safe alternatives to extend the shelf life of food products by preventing oxidation and contamination by pathogens that spoil food, meaning the sweet orange EOs can be considered as an innovative dual strategy for food preservation.	The main monoterpene was limonene (90.41%), myrcene (3.19%)	Could represent natural and safe alternatives to extend the shelf life of food products by preventing oxidation and contamination by food-spoiling pathogens, meaning that sweet orange EOs can be considered as an innovative dual strategy for food preservation.	(17)
5	Orange	Phytoactive chemicals found in *Citrus* oil can serve as prototypes in the production of new antimicrobial and antiparasitic drugs.	*In vitro*	*Citrus sinensis* Peel Oil Extraction and Evaluation as an Antibacterial and Antifungal Agent	The antibacterial potential of oil against bacterial strains, i.e., *E. coli*, *S. aureus* and *S. agalactiae* exhibited that the oil efficiently controls the bacterial growth. The highest zone of inhibition was observed against the strain *E. coli*. The antifungal potential of oil for pathogenic fungal strains, i.e., *A. flavus*, *A. niger*, *A. alternata* showed that the oil significantly inhibits fungal growth.	β-pinene (0.55%), limonene (96–98%), α-pinene (0.29%), myrcene (1.3–1.45%) and octanol (0.37–0.53%).	The antibacterial potential of the oil against bacterial strains, i.e., *E. coli*, *S. aureus* and *S. agalactiae*, demonstrated that the oil efficiently controls bacterial growth. The diameter of the inhibition zone was maximum against *A. flavus*. Phytoactive chemicals found in *Citrus* oil can serve as prototypes in the production of new antimicrobial and antiparasitic drugs.	(18)
6	Cinnamon	Significant decrease in fasting blood glucose, plasma C-peptide, serum triglyceride, total cholesterol, and blood urea nitrogen levels, with significant increase in high-density lipoprotein after 35 days. Glucose tolerance was improved and pancreatic islet β-cells showed increased immunoreactivity.	*In vivo* (Rat), STZ	Encapsulation of cinnamon oil in whey protein counteracts the disturbances in biochemical parameters, gene expression, and histological picture of the liver and pancreas of diabetic rats	Both doses improved glucose, insulin, SOD, GSH, amylase, lipid profile and hepatic MDA levels. Gene expression was modulated to favor antidiabetic results. Positive histological changes are observed in the liver and pancreas.	More than 86% of these volatiles: cinnamaldehyde (88.7%), 1,8 cineole (2.02%), acetic acid, 1,7,7 trimethylbicyclo [2.2.1] hept2yl ester (1.79%), α-Pinene (1.45%), and α-Terpineol (0.92%)	The protective role of COE against disturbance in biochemical parameters, antioxidant capacity, gene expression, and histological changes in the liver and pancreas in STZ-treated rats was evaluated. It was reported that STZ has a rapid action on pancreatic β cells and induces a massive reduction of these cells in islets of Langerhans resulting in hyperglycemia and the ROS generation in β cells following the increasing of ROS in other organs	(19)
7	Cinnamon	*Cinnamomum zeylanicum Blume* offers novel approach to chemotherapy treatment	*in vitro*	Optimization of Cinnamon (*Cinnamomum zeylanicum Blume*) Essential Oil Extraction: Evaluation of Antioxidant and Antiproliferative Effects	Cinnamon essential oil was able to remove hydrogen peroxide depending on the amount, 0.7 mg/mL of cinnamon essential oil exhibited 50% hydrogen peroxide removing activity.	cinnamaldehyde (77.34%), transcinnamyl acetate (4.98%), benzenedicarboxylic acid (3.55%), α-pinene (2.6%) and coumaric acid (1.79%)	Essential oils seem to be useful as possible antitumor agents. This work studied the effect of different concentrations of *Cinnamomum zeylanicum Blume* essential oil against HeLa (0.0625 to 4 μ g/mL) and Raji (0.375 to 12 μ g/mL) cell lines. mL	(20)
8	Coriander	The coriander oil and linalool combinations conferred a synergistic anthelmintic effect	*In vitro*	Nematocidal Effects of a Coriander Essential Oil and Five Pure Principles on the Infective Larvae of Major Ovine Gastrointestinal Nematodes *In Vitro*	The coriander oil and linalool showed the most inhibitory effects against L3s. Combined treatment using coriander oil enhanced with additional linalool offered more synergistic effect on larval motility indicated by the extensive cuticular damage of treated L3s.	linalool (68.03%), α-tinene (9.71%), γ-terpinene (10.48%) and camphor (11.76%)	The mode of action of coriander oil and linalool is related to disruption of membrane function	(21)
9	Coriander	Inhibition of 65 essential oils and 21 essential oil mixtures against several species/clinical strains of dermatophytes of two main genera, *Microsporum* and *Trichophyton*	*In vitro*	Activity of various essential oils against clinical dermatophytes of *Microsporum* and *Trichophyton*	All genera/species tested were completely inhibited for 21 days after a single application	The exact content of the individual components of each oil was not tested or evaluated	Inhibition of strain growth for a long time	(22)
10	Parsley	Parsley presented the best antioxidant profile	*In vitro*	Chemical Composition, Antioxidant and Antimicrobial Activity of Essential Oils from Organic Fennel, Parsley, and Lavender from Spain	Parsley had the highest phenolic content. Overall, parsley presented the best antioxidant profile, given its highest % of inhibition of DPPH radical (64.28%) and FRAP (0.93 mmol/L Trolox), but had a pro-oxidative behavior by TBARS.	myristicin (36.15%), apiole (20.97%), α-pinene (15.47%), and β-pinene (10.43%).	The antioxidant activity of organic fennel, parsley, and lavender EOs was assessed by evaluating hydrogen donating ability, or radical scavenging activity, using the stable radical DPPH.	(13)
11	Rosemary	Evaluation of Their Chemical Composition, Genotoxicity, Antimicrobial, Antiviral, and Antioxidant Properties	*In vitro*	Rosemary Extract and Essential Oil as Drink Ingredients: An Evaluation of Their Chemical Composition, Genotoxicity, Antimicrobial, Antiviral, and Antioxidant Properties	The essential oil in dilutions of up to 5% and the extract in the range of 25–90% are not genotoxic, and confer antiviral, antifungal, and antioxidant properties; the extract also confers important antibacterial properties, whereas the essential oil is mainly effective against *S. aureus*.	Monoterpenes (95.57%), sesquiterpenes amounted only to 4.24%	The antioxidant activity was evaluated by the DPPH radical method, the Ferric reducing antioxidant power (FRAP) assay and the ABTS assay and the radical scavenging activity among rosemary essential oils is mainly due to the different amounts of major compounds in the essential oils	(23)
12	Thyme	To determine the bioactive compounds, present in thyme essential oil and utilize the nanoemulsion technique to enhance its protective capacity against oxidative stress, genotoxicity, and DNA damage caused by biosynthesized titanium dioxide nanoparticles.	*in vivo*	Improvement of the antioxidant activity of thyme essential oil against biosynthesized titanium dioxide nanoparticles-induced oxidative stress, DNA damage, and disturbances in gene expression *in vivo*	The TEO nanoemulsion enhances the biological activity of the oil, improves its antioxidant properties, and provides protection against oxidative damage and genotoxicity induced by TiO2-NP.	The successful identification of 17 bioactive compounds was achieved in thyme essential oil (TEO), with thymol and carvacrol being the main components present in this essential oil.	Sixty male Sprague–Dawley rats were divided into six groups and orally treated for a period of 21 days. These groups included the control group, the TEO-treated group, the TEON-treated groups, the TiO2-NP-treated group, and those groups that received both TiO2-NP and TEO. Blood and tissue samples were collected for various analyses.	(24)

Table 2 shows how this essential oil affects health, the analysis of the major active constituent identified, and relevant results in the essential oils performed. The lemon essential oil has been found to contain a significant amount of limonene, which has strong antioxidant properties and can help eliminate DPPH radicals (16). Research has shown that it can also be effective as a food preservative, with concentrations of 0.06 and 0.312 mg/g potentially preventing the growth of pathogenic bacteria like *L. monocytogenes* (15). Limonene may also positively affect the immune system and intestinal microbiota (25). On the other hand, orange essential oil is also a natural citrus preservative in food due to its antimicrobial properties. Its main compounds, citral, and linalool, have been found to have superior effects on intestinal microbiota in mice when compared to lemon essential oil (17). It is important to note that further research is needed to fully understand the potential benefits of these essential oils in the areas mentioned. And findings regarding essential oils like parsley, cumin, coriander, thyme, and rosemary were relatively scarce, mostly showing effects on health. Mainly, parsley exhibits both antioxidant and prooxidative behavior, making it a promising subject for further investigation. Cumin, with volatile compounds inhibiting neutrophil activation, is strongly associated with treating inflammatory pathologies. Supplementation with cumin in patients with metabolic syndrome is estimated to decrease diastolic pressure (12). Coriander is known for its resistance to bacterial infections and tends to improve health in tilapia (10). Thyme demonstrated inhibition of cell proliferation, varying with the dose in all evaluated tumor cell lines (26).

Table 3 highlights the various omics mechanisms of the essential oils already mentioned above. The essential oils of lemon, orange, and cinnamon influence the intestinal microbiota in the same way the essential compounds of coriander inhibit the activity of gastrointestinal pathogenic bacteria. The essential compound of rosemary oil has an anti-Warburg effect on gastric carcinoma, dose, and time. And a thymol compound-dependent cytotoxic effect on cancer cell lines PC-3, DU145, MDA-MB-231, and KLN205.

**Table 3:** Essential oils and omics mechanism.

	Essential oil	Health action	Omics of the study epigenetics, microbiota, metabolomics, genomics	Omics mechanism of action	Type of study	Study name	Relevant results	Reference
1	Lemon	Intestinal limonin boosts immunity by optimizing macronutrient metabolism.	Microbiota	16S rRNA gene analysis revealed that intestinal microbial diversity (α-diversity and b-diversity) was significantly modified by limonin intervention	Gut microbiota in mice	The gastrointestinal fate of limonin and its effect on the intestinal microbiota in mice	Microbial species richness increased significantly with limonin treatment (limonene being its main active compound), while low diversity was observed in obese mice fed a high-fat, high-sugar diet.	(25)
2	Orange	Orange essential oil, limonene, linalool and citral decrease short-chain fatty acids in intestines.	Microbiota	The effect of essential oil, linalool and citral on immune organ index, IgM and IL-2 was not significant (*p* > 0.05). A significant increase (*p* < 0.05) of H + K + -ATPase activity, IgA, IgG, and IL-2 in the limonene group.	Gut microbiota in mice	Effects of orange essential oil on intestinal microbiota in mice	Orange essential oil could affect the intestinal microbiota of mice and improve the relative abundance of *Lactobacillus.* This indicated a more obvious effect of limonene in addition to linallool and citral, on intestinal bacteria, as well as changes in blood immune index and short-chain fatty acids in mice.	(27)
3	Rosemary	Rosmarinic acid inhibits Warburg effect in gastric cancer cells.	Genomics	Protein expression was determined by Western blot assay. Mouse xenograft models were established using MKN45 cells to evaluate the anti-Warburg effect on gastric carcinoma *in vivo*.	Gastric cancer cells	Anti-Warburg effect of rosmarinic acid through miR-155 in gastric cancer cells	Rosmarinic acid suppressed glucose uptake and lactate production and inhibited the expression of the transcription factor hypoxia-inducible factor-1α. Inflammation promoted the Warburg effect in cancer cells. As expected, it inhibited pro-inflammatory cytokines and inflammation-related microRNAs.	(28)
4	Rosemary	Rosemary extracts degrade androgen receptors and reduce prostate cancer cell viability.	Microbiota	Standardized rosemary extract decreases the expression of the androgen receptor which appears to be regulated by the expression of CHOP/GADD153	Two human prostate cancer cell lines, 22Rv1 and LNCaP.	Rosemary (*Rosmarinus officinalis*) extract modulates CHOP/GADD153 to promote androgen receptor degradation and decrease xenograft tumor growth	Significant modulation of endoplasmic reticulum stress proteins was observed in cancer cells. This biphasic response suggests that standardized rosemary extract may preferentially target cancer cells rather than “normal” cells. This study indicated that the main active compound was carnosic acid.	(29)
5	Thyme	Thyme essential oil demonstrates antiproliferative and cytotoxic activity against leukemia, breast and lung cancer cell lines.	Genomics	Detection of cytotoxic concentration range and detection of antiproliferative activity of essential oil	Malignant cell lines (MOLT-4, MCF-7 and H460).	Detection of cytotoxicity of *Thymus vulgaris L*. essential oil in brine shrimp nauplii and cancer cell lines	Thymol (main active compound) induced a dose-dependent inhibition of cell proliferation in all tumor cell lines. Dose-dependent toxicity confirmed that the brine shrimp lethality test is a suitable method for preliminary toxicity testing of *Thymus vulgaris L*. essential oil on tumor cell lines.	(30)
6	Thyme	Thymol oil induces apoptosis in cancer cells in a time and dose dependent manner.	Genomics	Determine the antiproliferative activity and apoptotic effect of thymol in prostate cancer (PC-3, DU145), breast cancer (MDA-MB-231) and lung cancer (KLN205) cell lines.	Prostate cancer, breast cancer and lung cancer cell lines	Rosemary (*Rosmarinus officinalis*) extract modulates CHOP/GADD153 to promote androgen receptor degradation and decrease xenograft tumor growth	The study showed the dose-and time-dependent cytotoxic effect of thymol on cancer cell lines PC-3, DU145, MDA-MB-231 and KLN205. Thymol significantly induced apoptosis in all groups in a dose-dependent manner.	(26)
7	Cinnamon	Cinnamon essential oil has antimicrobial and anti-inflammatory effects that protect against inflammatory bowel disease.	Microbiota	The antibacterial activity of cinnamon is attributed to the destruction of the bacterial membrane, modification of the lipid profile and inhibition of cell division. Suppressed the expression of TLR4, myeloid differentiation factor 88 and nuclear factor kinase.	Mouse model	Effect of cinnamon essential oil on gut microbiota in the mouse model of dextran sodium sulfate-induced colitis	Treatment with CEO (main compound Cinnamaldehyde (68.95%)) improved symptoms of DSS-induced colitis in mice, as demonstrated by a decrease in body weight loss and disease activity index scores.	(31)
8	Thyme	Thymol protects colon epithelial cells from oxidative DNA damage, while geraniol protects against DNA methylation damage.	Genomics	The genoprotective effects of essential oil compounds against oxidative and methylating damage were evaluated by the comet assay in HT-29 colorectal adenocarcinoma cells. Most of these were cytotoxic to HT-29 cells at 250 ppm or higher after 24 h of exposure.	The comet assay in HT-29 colorectal adenocarcinoma cells.	Effects of Essential Oil Compounds Against Oxidative and Methylated DNA Damage in Human Colon Cancer Cells	Thymol was the most protective compound against oxidative DNA damage and geraniol also protected cells against DNA methylation damage. Other notable compounds were nerolidol, geraniol, methyl isoeugenol, eugenol, linalool and a commercial mixture (Agolin).	(32)
9	Rosemary	*Rosmarinus officinalis* compounds like rosmarinic acid, carnosic acid and carnosol give it unique therapeutic effects and increase metabolite production.	Metabolomics	The identification and regulation of the biosynthetic pathways and genes will enable the large-scale production of these compounds	Metabolomics and transcriptomics data of *R. officinalis* were retrieved from the Microbial and Eukaryotic Systems Resource Database.	System network analysis of *Rosmarinus officinalis* transcriptome and metabolome—Key genes in biosynthesis of secondary metabolites	The core genes include copalyl diphosphate synthase (CDS), phenylalanine ammonia lyase (PAL), cineol synthase (CIN), rosmarinic acid synthase (RAS), tyrosine aminotransferase (TAT), cinnamate 4-hydroxylase (C4H) and MYB58 are responsible for the biosynthesis of important secondary metabolites.	(33)

## 5 Cinnamon

### 5.1 Health benefits

In a study by Mohammed et al. (19), six groups of male rats were treated orally for 4 weeks. The control and STZ-treated groups were compared with groups that received low or high doses of cinnamon oil emulsion (COE; 200 or 400 mg/kg Bw) and groups of STZ-treated rats that received COE in low or high doses. The results showed a significant decrease in fasting blood glucose levels, plasma C-peptide, serum triglycerides, total cholesterol, and blood urea nitrogen, with a substantial increase in high-density lipoproteins after 35 days. Glucose tolerance improved, and an increase in pancreatic islet β cells was observed. Both doses improved glucose, insulin, SOD, GSH, amylase, lipid profile, and hepatic MDA levels. Positive histological changes were also observed in the liver and pancreas. The current results revealed that cinnamon oil emulsion (COE) produced more than 1% of total volatile compounds, and GC/MS identified 16 compounds. More than 86% of these volatiles originated from 5 volatiles and included cinnamaldehyde (88.7%), 1,8 cineole (2.02%), acetic acid, 1,7,7trimethylbicyclo [2.2.1] heptyl ester (1.79%), α-Pinene (1.45%), and α-Terpineol (0.92%). This research aimed to examine the potential of essential oils as antitumor agents. *In vitro* models, including phosphomolybdenum, DPPH, and H2O2 methods, were used to achieve this. This study used BHT (butylhydroxytoluene) and ascorbic acid (vitamin C) as positive controls for comparison (20).

### 5.2 Food benefits

This review focuses on analyzing the effects of cinnamon oil when used as an additive in poultry feed, addressing its influence on various aspects such as bird performance, carcass characteristics, meat quality, its impact on cholesterol reduction, its antioxidant activity, its effects on immunity and considerations related to microbiology. The key results of this research indicate that including cinnamon essential oil extracts as additives in poultry feed carries notable benefits in terms of improved performance, reduced blood cholesterol levels, and increased activity: antioxidant, immunity booster, and favorable microbiological considerations. Cinnamon could represent a viable alternative to antibiotics, providing excellent safety in aspects related to animal health, the environment, and the economy in the poultry industry. Furthermore, it was observed that the main component of cinnamon oil is Cinnamaldehyde, which approximately represents Cinnamaldehyde (88.2%), eugenol (1.0%), and benzyl alcohol (8.0%) in its composition (34).

### 5.3 Actions in health

One of the potential health benefits is protection against colitis, which is an anti-inflammatory bowel disease. This is related to the mechanism of action, antimicrobial activity, and anti-inflammatory effect of cinnamon due to the alteration in the bacterial membrane, modifications in the lipid profile, and the inhibition of cell division, as shown in a mouse model study. *Cinnamomum osmophloem* reduced the expression of Toll-like receptor 4, myeloid adapter protein 88, and nuclear kinase in mice with colitis that had received endotoxin, suggesting an anti-inflammatory effect. In addition, it was identified that the predominant active component in cinnamon essential oil is cinnamaldehyde, which constitutes approximately 68.95% of its composition (31).

## 6 Cilantro

### 6.1 Health benefits

In a study conducted by Helal et al. (21) on the motility of third-instar larvae (L3) belonging to the Trichostrongylidaeindicate that these essential oils and their components, such as linalool (68.03%), α-tinene (9.71%), γ-terpinene (10.48%) and camphor (11.76%), could effectively combat infections caused by helminths.

Moreover, studies have shown that essential oils, including cilantro oil, can effectively hinder the growth of clinical strains of dermatophytes belonging to two primary genera, *Microsporum* and *Trichophyton*, for up to 21 days. It is worth noting that accurately identifying the specific individual is crucial (22).

### 6.2 Food benefits

The antioxidant, antimicrobial, and anti-biofilm properties of the essential oil obtained from cilantro (*Coriandrum sativum L.*) are the most studied concerning its possible application in food. The main volatile components identified in coriander essential oil in an *in vitro* study were mainly β-linalool, which accounted for 66.07% of the total content, showing a high antioxidant activity with an inhibition percentage of 51.05% in eliminating radicals. Its antibacterial activity presented the most effective against *B. subtilis,* followed by *S. maltophilia* and *Penicillium expansum* (9). An *in vivo* study assessed the effectiveness of adding 1% coriander oil to tilapia feed to enhance health and immunity against bacterial infections. The key compounds in this oil were linalool and geranyl acetate (10).

## 7 Cumin

### 7.1 Health benefits

Recent studies have shown that cumin oil can potentially suppress neutrophil activation, which could be beneficial in treating inflammatory diseases that involve high levels of neutrophil activity. This includes respiratory burst and degranulation induced by formylpeptide receptor agonists fMLF/CB and MMK1 in human neutrophils. These effects’ mean inhibitory concentration (IC50) ranges from 3.8 to 17.2 μg/mL. Additionally, it has been noted that cumin oil contains a high percentage of cumin aldehyde, which makes up 49.9% of its composition (11). Morovati et al. (12) found that cumin essential oil significantly reduced diastolic blood pressure in patients with metabolic syndrome after 8 weeks of treatment.

## 8 Parsley

### 8.1 Health benefits

Parsley essential oil exhibits an antioxidant profile due to its higher percentage of DPPH radical inhibition and FRAP value; however, it also showed pro-oxidative behavior according to the TBARS test. On the other hand, parsley essential oil demonstrates more significant bacterial activity after lavender. The main components were myristicin (36.15%), apiole (20.97%), α-pinene (15.47%), and β-pinene (10.43%). The presence of alkyltetramethoxybenzene, limonene and elemicin (6.45, 4.74 and 2.74%, respectively) was also relevant (13).

## 9 Lemon

### 9.1 Health benefits

It has been shown that the essential oil obtained by hydrodistillation of discarded lemon leaves contains enough chemicals can inhibit the growth of harmful microorganisms such as *C. albicans, L. monocytogenes*, and *S. aureus*. The oil’s most abundant components are limonene (with a concentration of 260.7 mg/mL), followed by geranial (102.6 mg/mL) and neral (88.3 mg/mL). The study also found that a concentration of 25 μM of the oil led to a significant reduction in cell viability, with a 33% decrease in HeLa cells and a 27% decrease in A375 cells. This was also accompanied by notable changes in cellular morphology (14).

The main components of lemon essential oil responsible for its antibacterial and antioxidant properties are terpenoids, with d-limonene being the most abundant. D-limonene exhibits the highest antioxidant capacity and effectively removes DPPH radicals (16).

### 9.2 Food benefits

In the food industry, lemon essential oil’s antioxidant and antimicrobial properties can be used as a preservative. According to Ben Hsouna et al. (15), the application of this oil at concentrations of 0.06 and 0.312 mg/g presents promising potential for the prevention of contamination and the development of pathogenic bacteria, especially *L. monocytogenes*, which opens new perspectives in this field.

### 9.3 Action in health

The effect of limonin on the intestinal microbiota has been investigated in mice, and a significant increase in the diversity of the microbiota present in the colon of mice fed limonin has been observed. Thus, it was highlighted that the composition of the intestinal microbiota community was different compared to the control group. A prediction was made that limonin would positively affect the regulation of amino acid metabolism, lipids, and immune system function. It is highlighted that it can significantly suppress diseases related to the immune system and markers of infectious diseases based on its influence on the intestine (25).

## 10 Orange

### 10.1 Health benefits

The *Citrus sinensis* peel oil has potential antibacterial, antifungal, and antiparasitic effects, according to a study conducted by Anwar et al. (18). The analysis showed that the maximum inhibition zone diameter was 14 mm against *E. coli*, and the minimum was 10 mm against *S. agalactiae*. Moreover, the oil was 60% effective in inhibiting leishmaniasis at a 50 μg/mL concentration after 48 h of incubation. The oil’s antimicrobial properties were also demonstrated, suggesting its possible use as a natural food preservative or an effective treatment against various pathogenic organisms. The oil contains β-pinene (0.55%), limonene (96–98%), α-pinene (0.29%), myrcene (1.3–1.45%), and octanol (0.37–0.53%).

### 10.2 Food benefits

A study assessed commercial orange essential oils’ chemical compositions, antioxidant, and anti-pathogenic properties [*Citrus sinensis (L.) Osbeck*]. The study used cold pressing (EOP) and cold pressing followed by steam distillation (EOPD). The analysis revealed that both essential oils contained a high percentage of monoterpene hydrocarbons, mainly limonene (89.8 to 90.4%) and myrcene (3.1 to 3.2%). Although both essential oils had similar reducing capacities, EOP showed a more remarkable ability to eliminate free radicals. Regarding anti-pathogenic properties, both essential oils inhibited the biomass and cellular viability of *Staphylococcus aureus* and *Pseudomonas aeruginosa* in their biofilms. Additionally, both methods effectively reduced the production of elastase, pyocyanin, and quorum-sensing autoinducers, particularly in Gram-negative bacteria. These findings indicate that EOP and EOPD demonstrate significant antioxidant and anti-pathogenic properties (17).

### 10.3 Action in health

A recent study examined the effects of administering orange essential oil, as well as limonene, linalool, and citral, directly into the stomachs of mice. The researchers were interested in understanding how these substances might affect the mice’s intestinal microbiota and biochemical parameters. The study found that all four substances could influence the mice’s intestinal microbiota composition, with the relative proportion of *Lactobacillus* increasing in response to treatment. However, the mice that received limonene had a notably different bacterial composition in their cecum and colon than the other groups. These findings suggest that limonene may have a more pronounced effect on intestinal bacteria, which can lead to significant changes in blood immunological markers and short-chain fatty acid levels in mice (27).

## 11 Thyme

### 11.1 Health benefits

Thyme essential oil (TEO) has health benefits due to its bioactive compounds. TEO nanoemulsion improves its biological activity and antioxidant properties and protects against oxidative damage and genotoxicity caused by TiO2-NP. TEO contains 17 bioactive compounds, with thymol and carvacrol being the main components (24).

### 11.2 Action in health

Thymol has been found to impact prostate cancer (PC-3), breast cancer (MDA-MB-231), and lung cancer cells. The antiproliferative activity of cancer cells varies depending on the dose and time of exposure. Thymol also significantly induces apoptosis in all groups, with the intensity of the response varying depending on the dose administered (26).

Likewise, the antiproliferative activity has been evaluated (*in vitro*) using three human tumor cell lines: MCF-7 (breast adenocarcinoma), H460 (lung carcinoma), and MOLT-4 (acute lymphoblastic leukemia) using the MTT assay, demonstrating a dose-dependent inhibition of cell proliferation in all tumor cell lines evaluated, and differential sensitivity between them. The main components of the essential oil included thymol (36.7%), p-cymene (30.0%), γ-terpinene (9.0%), and carvacrol (3.6%) (30).

Understanding oils’ effects on our health is crucial, particularly in preventing cellular toxicity and genotoxicity. A recent study found that essential oil compounds can offer protective benefits against oxidative and methylating damage, as seen through comet assays on colorectal adenocarcinoma HT-29 cells. While most of these compounds were found to be cytotoxic to HT-29 cells, they only reached cytotoxic levels at doses equal to or greater than 250 ppm after exposure for 24 h. The study identified thymol as the most effective component in protecting DNA against oxidative damage, while geraniol also showed promise in protecting against DNA methylation damage. This research highlights the potential of essential oil compounds, especially thymol, in protecting the colonic epithelium against oxidative DNA damage and geraniol against DNA methylation damage (32).

## 12 Rosemary

### 12.1 Health benefits

A study by Christopoulou et al. (23) assessed the chemical composition, genotoxicity, and antimicrobial, antiviral, and antioxidant properties of certain substances. The results showed that the essential oil, at concentrations of up to 5%, and the extract, ranging from 25 to 90%, did not exhibit any genotoxic effects. The essential oil and the extract also demonstrated antiviral, antifungal, and antioxidant properties. Specifically, the extract exhibited notable antibacterial properties, while the essential oil was primarily effective against *S. aureus*. The essential oil mainly comprised monoterpenes, constituting 95.57%, whereas sesquiterpenes only represented 4.24%.

### 12.2 Action in health

A study on rosmarinic acid (RA) and the anti-Warburg effect in gastric carcinoma suggested that rosemary oil may reduce glucose uptake and lactate production in cancer cells. It was found that RA inhibits the expression of hypoxia-inducible factor 1α, which is involved in the glycolytic pathway. In cancer cells, inflammation promotes the Warburg effect. However, RA reduces the production of pro-inflammatory cytokines and inflammation-related microRNAs, which suggests that RA could suppress the Warburg effect through an inflammatory pathway related to interleukin (IL)-6 and the transcription factor STAT3 (28).

Using essential oil to improve the functioning of the endoplasmic reticulum can be a helpful health action in reducing the viability of prostate cancer cells and promoting the degradation of androgen receptors. A study evaluated the effects of rosemary extract, standardized in carnosic acid, on two types of human prostate cancer cells, 22Rv1 and LNCaP, and prostate epithelial cells collected from two patients undergoing radical prostatectomy. The study found that cancer cells significantly altered endoplasmic reticulum stress proteins, while normal prostate epithelial cells did not suffer endoplasmic reticulum stress. This two-stage response suggests that standardized rosemary extract might be a preferable treatment option for cancer cells rather than normal cells.

On the other hand, some results suggest that the identified core genes, such as copalyl diphosphate synthase, phenylalanine ammonia-lyase, cineole synthase, rosmarinic acid synthase, tyrosine aminotransferase, cinnamate 4-hydroxylase, and MYB58, could play a crucial role in the metabolism of *Rosmarinus officinalis*. This genetic analysis provides valuable information for genetic and metabolic engineering research to improve the biosynthesis of secondary metabolites in *Rosmarinus officinalis* (33).

## 13 Discussion

This review emphasizes the significant advancements of essential oils in various fields, such as medicine and the food industry, achieved through *in vitro*, *in vivo*, and cell line studies. These breakthroughs provide valuable insights that support the continuation of research in these areas. For instance, the study evaluates the impact of rosemary essential oil extract on reducing prostate cancer cells (28). Moreover, the effects of cinnamon oil as a food additive for poultry are examined. The study explores its influences on avian performance, meat quality, cholesterol reduction, antioxidant activity, and immunity (35).

Insufficient literature exists on essential oils concerning innovative omics action mechanisms, more relevant applications in food to ensure quality, and their benefits and impact on health. Therefore, it is recommended that future research should conduct studies using cell lines (*in vivo*). This approach promotes more natural additives, thereby reducing the intake of processed additives that may adversely affect human health in the long run. These limitations are significant as they restrict understanding of essential oils’ potential benefits to improve the quality of life and address specific health-related needs in the food industry.

The potential health benefits of rosemary oil have been extensively studied and documented. Compounds included in rosemary oil, notably oleic and linoleic acid, are known to benefit cardiovascular health (36). Evidence is limited for the effects of rosemary oil on glucose. Further research, with more rigorous experimental designs and in larger populations, is required to reach more definitive conclusions about these properties (35, 37–39). One of the main factors contributing to rosemary oil’s potential usage in pharmacological and biotechnological applications is its biological qualities (8, 40). For example, natural antioxidants such as rosemary extracts have shown high thermal resistance and superior antioxidant activity compared to synthetic antioxidants (41). This makes them beneficial for preserving the quality of oils, particularly in high-heat cooking processes like frying (42, 43). Beyond acting as food preservatives, rosemary oil components may have direct health advantages. The oil’s anti-inflammatory effects may also promote brain health by preventing neuronal cell damage (44–46). Ongoing research reveals rosemary oil’s multipronged therapeutic potential—from enhancing heart health to shielding brain function to inhibiting tumor growth (47–49).

Several studies have been conducted that provide convincing evidence of the usefulness of cinnamon essential oil, but most are *in vitro* or animal research. More large-scale, high-quality human clinical trials are still needed to confirm the effects, establish the optimal dose, assess long-term safety, etc. Mechanistic studies have elucidated certain pathways, such as modulation of glucose transporters, antioxidant enzymes and inflammatory markers that underlie the observed effects. However, further work is needed to elucidate the full pharmacokinetic and pharmacodynamic profile, especially of key actives such as cinnamaldehyde (50–53).

Most research has focused specifically on cinnamon essential oil or cinnamaldehyde-standardized bark extracts. Comparisons of potency, bioavailability, and synergies between components require further research. Despite promising gastrointestinal effects, more extensive studies through rigorous randomized controlled trials on outcomes such as ulcerative colitis, irritable bowel syndrome, and dysbiosis are needed before clinical use can be recommended (54, 55). Safety, drug interactions, and contraindications have yet to be fully established.

Some studies demonstrate the efficacy of coriander oil against organisms such as fungi and helminths and agree that linalool e is the chemically essential compound (56). However, other chemotypes and oil compositions should also be examined, e.g., those richer in decanal, borneol, or geranyl acetate for which these comparative studies would demonstrate bioactivity that may reveal differential effects (57, 58). In addition, more detailed analyses, especially in food matrices exploring lipid peroxidation, protein oxidation, effects on shelf life, etc., would provide additional information along with limited safety studies (59–61). Toxicity, pharmacokinetic, and residue level studies in food-producing animals could allow the administration of higher standardized doses to enhance health effects (62–65). Finally, few *in vivo* studies explore the bioavailability, metabolism, and excretion of key actives such as linalool and the impact of long-term repeated dosing. Such pharmacokinetic data can help correlate *in vitro* and clinical results (64, 66).

Finally, lemon essential oil, the main component, D-limonene, boosts antimicrobial and antioxidant effects (15, 67, 68). However, further studies are needed on possible synergies with other components such as geranial (69, 70). In addition, rigorous human clinical trials are required to validate efficacy as a natural food preservative and to determine optimal dosage (71–73). The effect on the gut microbiome is promising, but it is not yet clear what specific changes in bacterial composition drive the observed health outcomes (68, 74).

## 14 Conclusion

Essential oils extracted from plants have been valued for their medicinal properties for centuries. Modern scientific research is now unraveling the composition and bioactivity of these complex natural extracts, providing insights into how essential oils might be used to help promote health and well-being. Based on the evidence summarized in the provided documents, several key conclusions regarding cinnamon, cilantro, cumin, parsley, lemon, orange, thyme, and rosemary essential oils can be drawn.

Firstly, these essential oils demonstrate varying degrees of antioxidant, antimicrobial, anti-inflammatory, or other beneficial biological effects. These effects are mediated by bioactive phytochemicals in the oils, such as cinnamaldehyde, linalool, limonene, thymol, and carnosic acid. The impacts of oils depend not just on their chemical composition but also on factors like genetics and growth conditions of the source plants. Standardization and quality control are thus important when studying and applying essential oil preparations. Secondly, many oils show promise as natural food preservatives—for example, inhibiting foodborne pathogens *like Escherichia coli* and *Staphylococcus aureus*. If efficacious and safe, plant-derived antimicrobials could replace synthetic additives. Areas needing more research include determining effects on food quality and nutritional content during storage.

Some of the main health benefits supported by current evidence are cinnamon oil improving glucose control and blood lipid levels; cilantro oil having parasite-killing properties; cumin oil reducing blood pressure; thyme oil protecting against oxidative cell damage and DNA mutations; and rosemary and thyme oils inhibiting cancer cell proliferation and viability. Further mechanisms of action are being elucidated—for instance, rosemary compounds may suppress altered metabolic pathways in tumor cells. More clinical trials are warranted to verify therapeutic efficacy and safety. An emerging area of research is how essential oil components like limonene and thymol influence intestinal microbiota populations.

So, essential oils are promising candidates for health promotion and disease treatment. However, converting traditional claims into evidence-based applications requires meticulous methodology. Key priorities in the future are clinical evaluations demonstrating efficacy, standardization, and quality control of oil preparations, untangling oils’ mechanisms of action, and further analyzing effects on human microbiota. With rigorous science illuminating their real therapeutic potential, essential oils could reveal themselves to be far more than just pleasant natural scents.

### 14.1 Limitations

This review is limited by the variability in the chemical composition of essential oils since these can vary according to the species/variety of the plant, growing conditions, time of harvest, extraction method, etc., making standardization and generalization of results difficult. Likewise, there is little information on the absorption, distribution, metabolism, and excretion (pharmacokinetics) of the components of essential oils in humans, and finally, there is not enough scientific evidence on interactions of essential oils with medications, nutrients, and other compounds - nutrient and drug interactions. For data analysis, the use of Cochrane tools to assess the risk of bias in randomized controlled trials, while for observational studies, the quality assessment tool of the US National Institutes of Health was not applied to the results since the included studies are *in vitro*.

## Author contributions

CP-O: Conceptualization, Validation, Data curation, Investigation, Writing – original draft. FG: Conceptualization, Data curation, Investigation, Writing – original draft, Formal analysis, Methodology, Software. CM: Data curation, Formal analysis, Investigation, Methodology, Writing – review & editing. JM: Funding acquisition, Project administration, Supervision, Validation, Writing – review & editing. AO-M: Funding acquisition, Project administration, Supervision, Validation, Writing – review & editing, Conceptualization, Methodology.
